# Comparative Study on Extrusion 3D Printing of Solid Propellant Based on Plunger and Screw

**DOI:** 10.3390/ma18040777

**Published:** 2025-02-10

**Authors:** Shixiong Song, Jiawei Shi, Quanbin Ren, Kai Miao, Min Tang, Hongbin Shi

**Affiliations:** 1 School of Astronautics, Northwestern Polytechnical University, Xi’an 710072, China; 2 The Institute of Xi’an Aerospace Solid Propulsion Technology, Xi’an 710025, Chinashihongbin8171@163.com (H.S.); 3 Academy of Aerospace Solid Propulsion Technology, Xi’an 710025, China; 4 School of Mechanical Engineering, Xi’an Jiaotong University, Xi’an 710049, China

**Keywords:** solid propellant, 3D printing, plunger extrusion, screw extrusion, computational simulation, comparative study, propellant fabrication

## Abstract

Extrusion-based 3D printing holds great potential for manufacturing solid propellants. Among the various methods, screw- and plunger-based extrusion are the most frequently reported techniques for propellant 3D printing, each employing different extrusion mechanisms. This paper compares the flow characteristics of these two methods through a combination of simulations and experiments. Simulation results reveal that propellant slurry in a plunger extrusion device exhibits relatively stable flow characteristics, especially near the nozzle outlet, with high flow velocity, high shear rate, and low-pressure distribution. Compared to the screw-based device, the plunger extrusion achieves a more uniform outlet velocity. In contrast, the screw extrusion device produces more complex rheological behavior, with backflow observed in the gap between the screw and the extrusion channel wall. However, the average pressure in the flow channel for screw extrusion (3885.11 Pa) is notably lower than that of plunger extrusion (7292.92 Pa). Experimental results indicate that the printing quality of plunger extrusion is comparable to that of screw extrusion. These findings provide valuable insights into advancing extrusion-based 3D-printing processes for solid propellants.

## 1. Introduction

Solid propellants serve as the primary thrust source for solid rocket engines, forming the foundation of solid rocket propulsion technology. Their performance quality directly affects the survivability and operational efficiency of both strategic and tactical missile systems, underscoring their growing importance in national defense and civilian applications [1,2]. However, as weaponry and equipment advance, and future combat scenarios become more complex and unpredictable, there is an increasing demand for enhanced solid propellant performance [3,4,5]. These advanced performance requirements often necessitate heterogeneous charge structures with complex geometries or integrated multimaterial formations. Traditional manufacturing methods, such as casting, are inadequate for producing propellant grains with these complex configurations [6,7]. In contrast, 3D printing, which utilizes advanced technology to rapidly and accurately fabricate components of virtually any complexity [8,9,10,11], has been widely applied and developed in the field of energetic materials across various countries. This approach has achieved promising results, confirming the feasibility of 3D printing for producing energetic materials such as explosives, propellants, and pyrotechnics [12,13].

In 2018, Chandru et al. [14] from the Indian Institute of Science successfully fabricated composite solid propellant grains with various complex geometries using Direct Ink Writing (DIW) technology. Their work demonstrated that propellant grains with controllable burn rates could be achieved by printing structures with controlled porosity levels. Subsequently, Kebede et al. [15] in India expanded on Chandru et al.’s research by employing an infrared heater to apply radiant energy to each printed layer, partially curing it to ensure sufficient strength and adhesion between layers. This approach helped maintain the structural integrity of the printed propellant grain. Meanwhile, McClain et al. [16] at Purdue University designed a UV-curable propellant slurry with a high solid content of 85 wt%, investigating the curing characteristics of light-curable composite propellants and confirming that the combination of UV curing with DIW could produce fully dense propellants. Additionally, Gunduz et al. [17] from Purdue proposed a mechanism for reducing nozzle flow resistance under high-amplitude ultrasonic vibration, specifically for highly viscous polymer materials. They demonstrated that introducing high-amplitude ultrasonic vibration in the nozzle created sufficient inertial force to significantly reduce wall friction and flow stress, effectively solving the problem of nozzle clogging in extrusion-based 3D printing. Building on this, McClain et al. [18] later prepared two types of high-solid-content (85 wt%) propellant slurries, one thermally curable and the other light-curable, using an ultrasonic printing nozzle. After curing, the resulting propellant grains exhibited significantly lower porosity and more compact, intact structures compared to those produced by traditional casting methods. Through extensive development, 3D-printing technology for solid propellants, especially extrusion-based approaches, has been thoroughly validated. Due to its procedural similarity to propellant casting, extrusion-based 3D printing is regarded as a highly promising process for fabricating complex geometries and even integrated multimaterial propellant grains.

Extrusion 3D printing involves extruding a propellant slurry of a certain viscosity through a nozzle using pneumatic pressure, a plunger, or a screw mechanism [19,20]. By controlling the movement of the nozzle and substrate, the propellant grain structure is directly deposited onto the substrate. Among these methods, pneumatic extrusion has shown limitations in printing accuracy due to factors such as driving force hysteresis and low controllability during printing [21,22]. Therefore, plunger- and screw-based extrusions have become the main methods for the 3D printing of propellants. Current research on propellant 3D printing largely focuses on the slurry properties, which must meet essential rheological requirements such as appropriate viscosity, high shear, compressive yield stresses [23,24,25], and controllable viscoelasticity, as well as a high solid content to ensure high energy output in the final propellant. Different extrusion methods significantly affect the quality of propellant printing; however, limited research has been conducted to compare and evaluate these extrusion methods.

This study investigates the rheological properties of custom-developed solid propellant slurries for light-curable-assisted extrusion 3D printing. Subsequently, the flow characteristics of the propellant slurry during plunger- and screw-based extrusion were compared through simulations, and experimental comparisons were performed to evaluate the performance of samples printed using both extrusion techniques. The findings provide guidance for selecting extrusion methods and optimizing the extrusion 3D-printing process for solid propellants.

## 2. Materials and Methods

### 2.1. Material Preparation

In this study, a substitute material for the solid propellant was created by adding a light-curable binder into the conventional solid propellant formula [26,27]. Table 1 below outlines the components and their respective functions.

The use of graded solid fillers with varying particle sizes promotes effective dispersion within the propellant formula. Table 2 below lists the proportions of these fillers.

**Figure 1:**
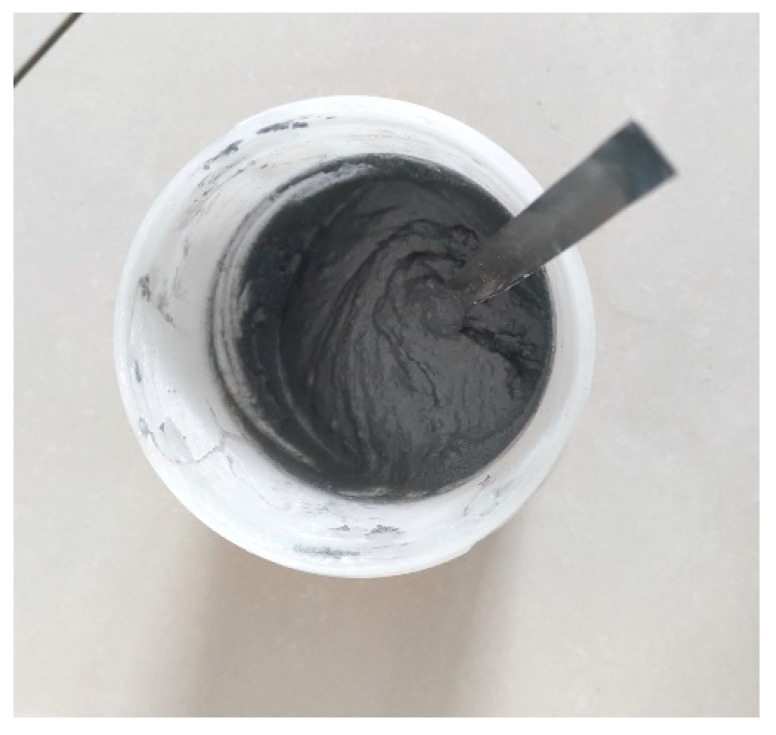
Schematic of propellant slurry.

After weighing, the materials were dispersed using a non-intrusive material homogenizer (ZYMC-180HV, Shenzhen Zhongyi Technology Co., Ltd, Shenzhen, China) for 5 min at 1000 rpm. They were then subjected to vacuum treatment for 1 h to obtain a uniformly mixed composite solid propellant slurry, as shown in Figure 1.

### 2.2. Testing and Evaluation

**Figure 2:**
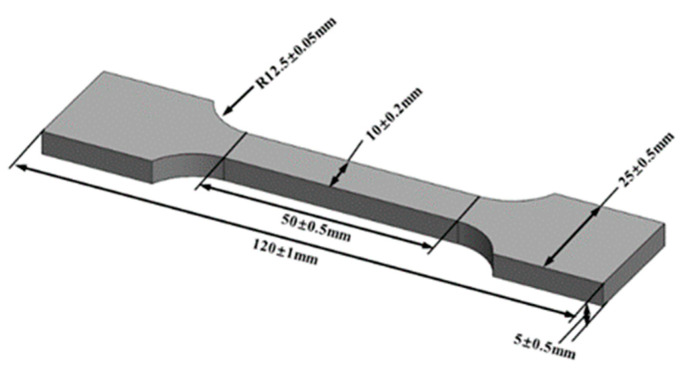
Dimensions of dumbbell-shaped tensile test specimen.

The rheological properties of the propellant paste were tested at room temperature (25 °C) using a rotational rheometer (MCR302, Anton Paar, Graz, Austria). A parallel plate geometry with a diameter of 25 mm and a 1 mm gap was selected, with shear rate measurements conducted over a range of 0.01–200 s⁻^1^. To compare the micromorphology of propellants printed using plunger and screw extrusion methods, field emission scanning electron microscopy (FE-SEM, MIRA II LMH, Tescan, Brno, Czech Republic) was employed to analyze the cross-sectional morphology of printed samples, allowing for detailed observation of pores and their distribution. Additionally, micro-computed tomography (YXLON, FF20 CT, Hamburg, Germany) was used to examine the distribution of internal defects within the samples. To assess the mechanical properties of propellant samples printed using plunger and screw extrusion, tensile tests were conducted at 20 °C using a universal tensile testing machine (INSTRON 5969, Instron, Norwood, NY, USA) with a crosshead speed of 2 mm/min. The tensile test specimens were dumbbell-shaped (Figure 2) with parallel-section dimensions of 10 mm × 5 mm. Each group of experiments printed 6 specimens, and we used 3M P240 sandpaper and referred to the dimensions and tolerance requirements provided in Figure 2 to perform the sanding process, with no visible surface defects before being tested. Each set of tensile tests ensured that there were at least 3 sets of relatively consistent experimental data. In addition, cylindrical grain configurations with a concentric filling pattern were chosen as the method for forming the samples, in order to minimize defects caused by path planning and to facilitate a more straightforward and intuitive comparison of the forming effects between the screw and plunger methods.

## 3. Results

### 3.1. Rheological Property Testing

**Figure 3:**
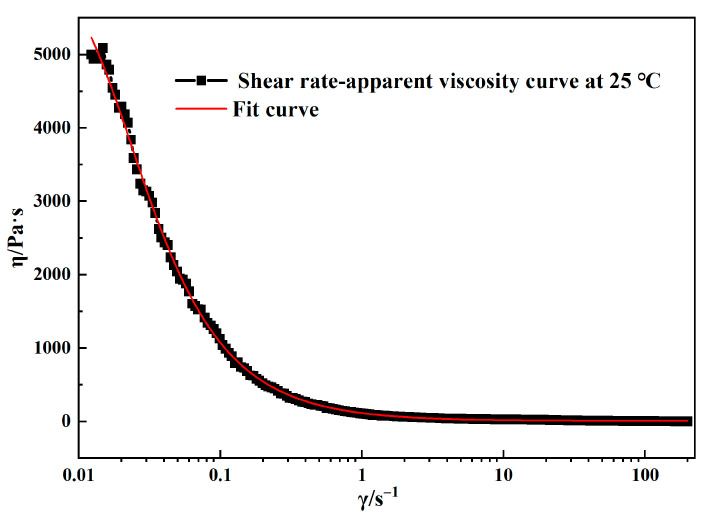
Curve representing change in propellant slurry viscosity with shear rate.

Using a rotational rheometer, a curve representing the variation in viscosity of the surrogate material with respect to shear rate was obtained, as shown in Figure 3. In a steady-state flow environment, the Carreau constitutive model effectively captures the relationship between shear rate and viscosity for this material. Specifically, the Bird–Carreau model is well suited for describing rheological properties across a broad range of shear rates [28]. The fitted curve for the surrogate solid propellant material aligns closely with the experimental data, showing a correlation coefficient of 0.9928. Therefore, the Bird–Carreau model was selected for subsequent simulations to represent the rheological properties of the propellant slurry. The fitting function is provided in Equation (1):

(1) $$\mu =\mu_{\infty }+(\mu_{0}-\mu_{\infty }){\left[1+{\left(\lambda \gamma \right)}^{2}\right]}^{\frac{n-1}{2}}$$

where *μ* represents the apparent viscosity of the slurry (Pa·s), *μ*_∞_ represents the infinite shear viscosity (Pa·s), *μ*_0_ represents the zero-shear viscosity (Pa·s), *λ* represents the relaxation time(s), and *n* is the power-law index. The values used in the equation are *μ*_∞_ = 0, *μ*_0_ = 6857.3, *λ* = 81.76, and *n* = 0.22.

### 3.2. Physical Model and Mesh Generation

**Figure 4:**
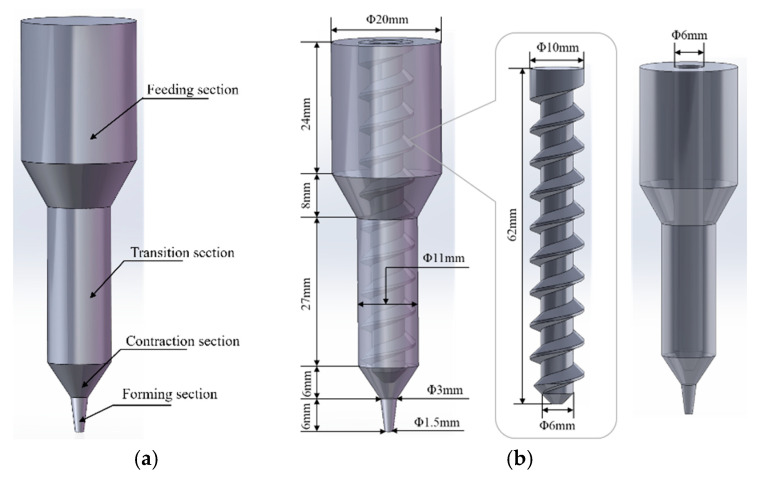
Three-dimensional model of extrusion structure including runner model of (**a**) plunger-based printer and (**b**) screw-based printer.

The extrusion process of the propellant slurry was simulated using ANSYS/POLYFLOW 2022R1 software. Figure 4 presents detailed dimensional models of the 3D printer extrusion mechanisms, featuring both plunger and screw structures. In the diagram, the flow channels for both mechanisms share identical external dimensions and are divided into feeding, transition, converging, and die sections from top to bottom.

**Figure 5:**
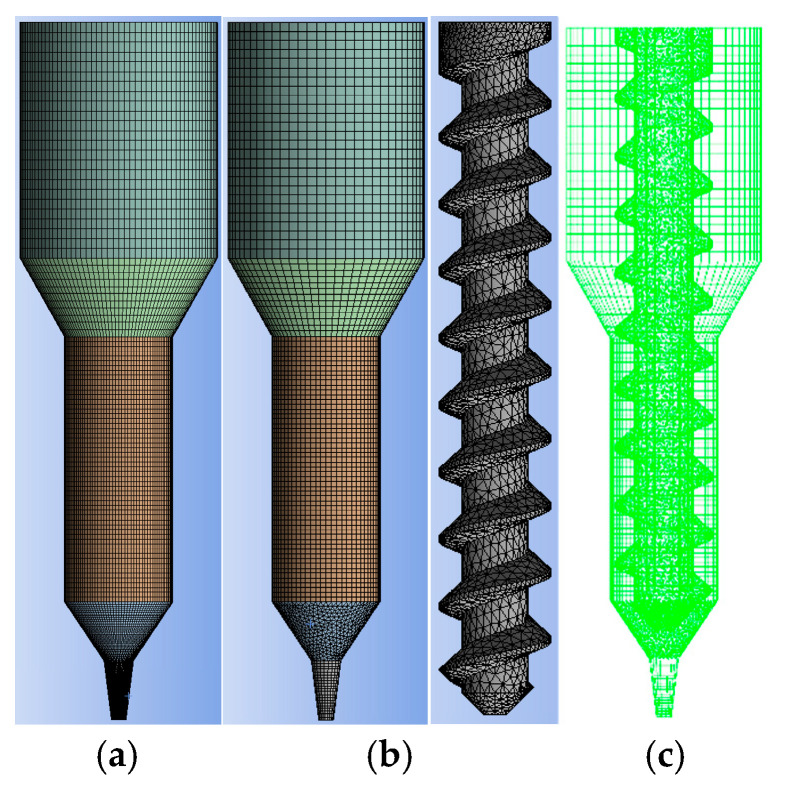
Mesh of model including (**a**) runner model of plunger-based printer after meshed, (**b**) runner model of screw-based printer after meshed, and (**c**) combined mesh.

Mesh results are shown in Figure 5. Since the middle region of the flow channel in the screw extrusion structure is continually covered by moving parts, preventing slurry extrusion flow in that area, this region was removed during simulation modeling to reduce the mesh element count and expedite computation. The dimensions of the removed regions were adjusted to match the root diameter of the screw. Additionally, a mesh overlay technique was adopted to combine the flow channel and screw meshes in the screw extrusion mechanism. This approach yielded a reliable and user-friendly mesh configuration, avoiding the need to create complex meshes for the interacting regions.

### 3.3. Boundary Condition Setting

Based on actual conditions, boundary conditions for the propellant slurry within the extrusion mechanism were established, covering inlet, outlet, and wall conditions. These boundary conditions primarily involve configuring the inlet and outlet parameters as well as defining flow variable values at the boundaries, as shown in Table 3. Specifically, the plunger inlet boundary was set as a free-flow inlet, while the screw mechanism required defining the moving part, with the inlet configured as a pressure inlet.

Composite solid propellant slurry is a typical non-Newtonian fluid [29], with viscosity that varies according to shear rate changes. A comparative analysis of the extrusion behavior of the plunger and screw mechanisms was conducted at a constant outlet flow rate of Q = 2.5 × 10^−8^ m^3^/s. Table 4 below provides the calculated parameters of the moving parts for both the plunger and screw [28,29,30] at this specified outlet flow rate.

## 4. Results and Discussion

### 4.1. Comparative Analysis of Velocity Distribution

**Figure 6:**
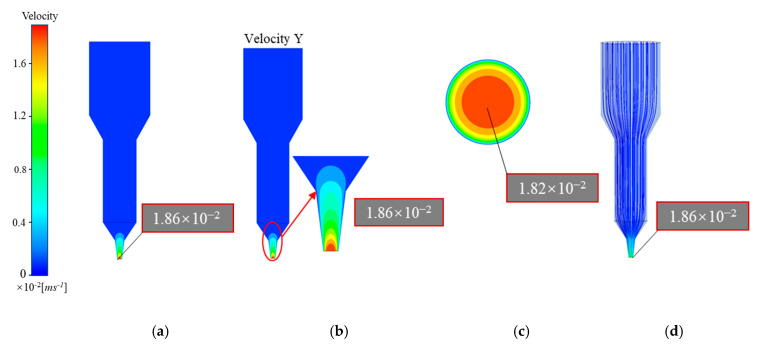
Velocity distribution of plunger extrusion structure: (**a**) overall velocity contour; (**b**) longitudinal velocity contour; (**c**) exit cross-section of velocity contour; (**d**) longitudinal velocity field.

**Figure 7:**
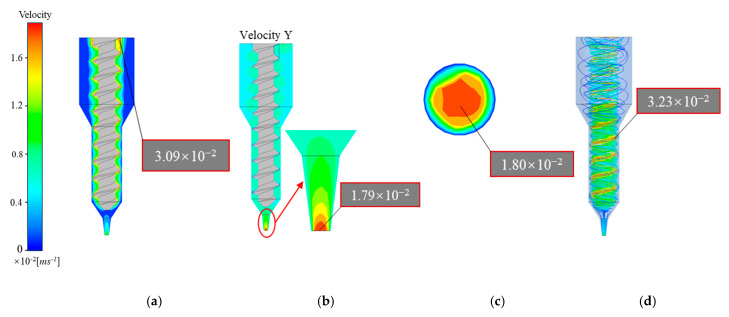
Velocity distribution of screw extrusion structure: (**a**) overall velocity contour; (**b**) longitudinal velocity contour; (**c**) exit cross-section of velocity contour; (**d**) longitudinal velocity field.

Figure 6a and Figure 7b show that the overall and longitudinal velocity fields in the flow channel of the 3D printer with a plunger structure remain largely consistent. However, as shown in Figure 7a,b, the screw-based flow channel exhibits notable variations between the overall and longitudinal velocity fields. The plunger structure achieves laminar flow from top to bottom, resulting in stable extrusion with minimal radial velocity distribution. In contrast, the screw structure generates rotational flow, a more complex extrusion process where the rotation of the screw drives the slurry downward, introducing a radial velocity component. This results in a more intricate velocity field distribution in the screw structure compared to the plunger structure.

As observed in Figure 6b and Figure 7d, the propellant slurry in the plunger structure exhibited velocities of approximately 0–1.86 mm/s within the barrel, transition, and upper converging sections of the flow channel. In these areas, the flow velocity remained low and evenly distributed. Beginning at the end of the nozzle’s converging section, the slurry streamlines gradually converged toward the center of the flow channel, and the fluid velocity began to increase, reaching a peak of 18.57 mm/s at the nozzle outlet. Additionally, slurry velocity was higher near the center of the nozzle outlet and lower near the nozzle boundary (Figure 7c), indicating an uneven velocity distribution across the outlet cross-section. High extrusion speeds during printing affect the consistency of cross-sectional velocity in the extruded filament, which in turn impacts the printing accuracy of the propellant grains.

Figure 7a,b display contour plots of the overall and longitudinal velocity fields within the screw-based printer flow channel, revealing noticeable velocity differences between these two regions. Near the screw, the slurry flows more rapidly, with relatively high speeds in the transition section. However, in the nozzle and barrel sections further from the screw, the slurry exhibits significantly lower velocities, approaching 0 mm/s compared to the area surrounding the screw. Additionally, the slurry experiences high longitudinal velocities near the screw threads but experiences backflow in the gap between the threads and the wall within the transition section. This backflow may lead to collisions and friction between the solid fillers in the slurry, introducing potential safety risks. Notably, a vortex forms at the top of the nozzle in the screw-based extrusion mechanism, as shown in Figure 7d. While this vortex aids in smooth slurry extrusion, it also intensifies velocity fluctuations, compromising outlet velocity uniformity.

**Figure 8:**
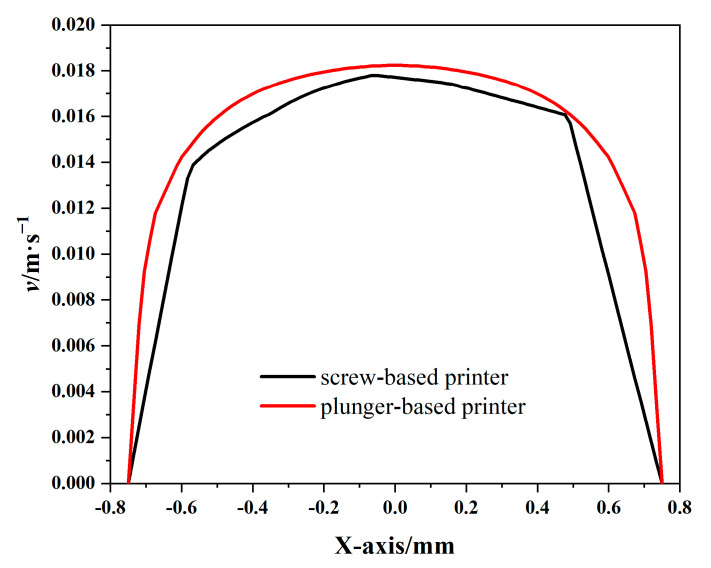
Curve of exit section velocity comparison.

As shown in Figure 6c and Figure 7c, the axial velocity at the outlet cross-section of the plunger extrusion structure exhibits superior uniformity and consistency compared to the screw extrusion structure. By examining the velocity variation curve along the *X*-axis at the outlet cross-section (Figure 8), it is apparent that the plunger structure exhibits an approximately parabolic velocity distribution. This velocity variation is more continuous than the stepped changes seen in the screw structure, thus better maintaining the uniformity of the extruded slurry’s velocity.

To accurately evaluate and characterize the velocity uniformity of the two extrusion structures, this study applied the velocity uniformity index, defined by Equation (2) [24], to quantitatively analyze the velocity uniformity at the outlet cross-section of each structure. By randomly selecting 100 velocity points at the outlet cross-section and calculating the values using Equation (2), it was determined that the velocity uniformity index for the plunger-based printer was 0.9276 compared to 0.8293 for the screw-based printer. Therefore, the plunger extrusion structure demonstrated superior outlet velocity uniformity over the screw structure, indicating its greater suitability for meeting higher printing accuracy requirements.

(2) $$\gamma_{v}=1-\frac{1}{2n}\sum_{i=1}^{n}\frac{\sqrt{{\left(v_{i}-\bar{v}\right)}^{2}}}{\bar{v}}$$

Here, *γ_v_* is the velocity uniformity index, ranging from 0 to 1, with higher *γ_v_* values indicating better flow uniformity. n represents the number of velocity measurement points. $v_{i}$ is the velocity at each measurement point (m/s). $\bar{v}$ is the average cross-sectional velocity (m/s).

### 4.2. Comparative Analysis of Pressure Distribution

**Figure 9:**
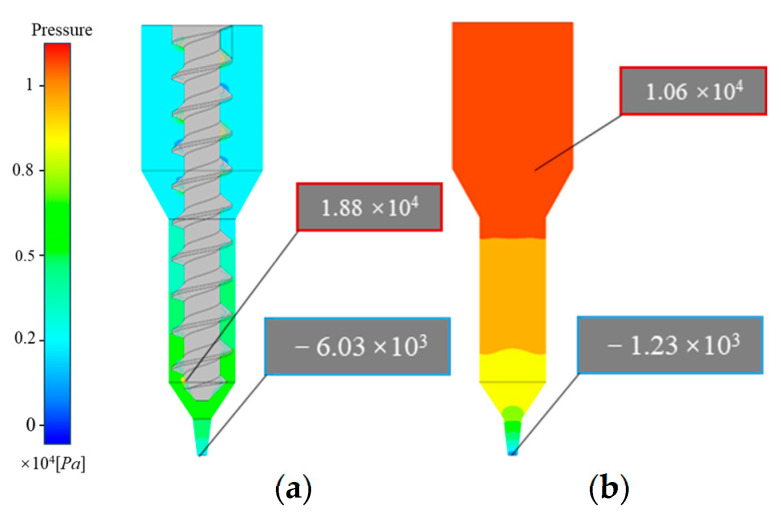
Pressure distribution contour: (**a**) screw-based printer; (**b**) plunger-based printer.

**Figure 10:**
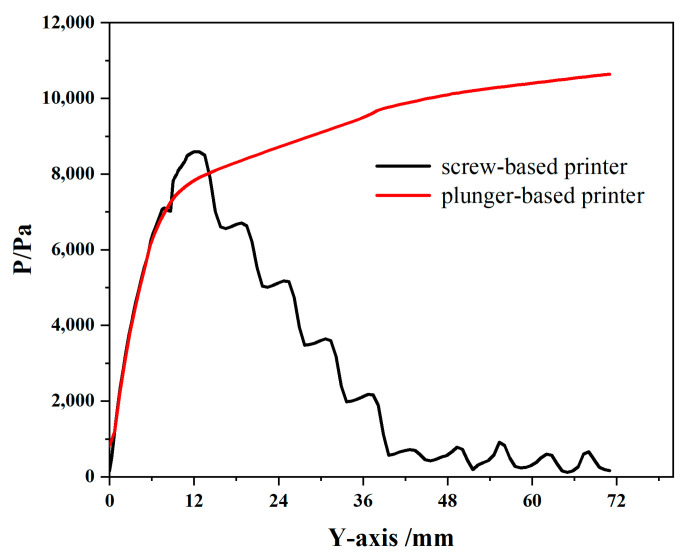
Curve of pressure comparison.

Figure 9a and Figure 10 illustrate the relatively complex pressure distribution within the screw extrusion structure. In this structure, the pressure along the flow channel initially rises and then decreases, with a sharp pressure drop near the outlet end. The highest pressure occurs in the nozzle contraction section. Additionally, negative pressure values are observed above the screw threads within the flow channel (Figure 9b), creating a pressure gradient with atmospheric pressure that allows continuous slurry feeding from the barrel. The maximum pressure in the nozzle contraction section of the screw extrusion mechanism is 8592.2 Pa, indicating a risk of slurry backflow if the nozzle pressure becomes excessive. Furthermore, pressure contours on the wall of the flow channel and the screw surface show that the screw experiences a higher pressure than the pipe wall, but it is significantly less than the fluid pressure within the extrusion tube (Figure 9a). The average pressure within the screw extrusion mechanism’s flow channel (3885.11 Pa) is substantially lower than that in the plunger extrusion mechanism (7292.92 Pa).

In contrast, as shown in Figure 9b and Figure 10, the pressure distribution within the plunger extrusion flow channel is relatively simple. The slurry pressure gradually decreases along the flow channel, with a rapid drop at the nozzle outlet, forming a pressure gradient that facilitates smooth slurry extrusion between the barrel and nozzle. Since the plunger supplies the extrusion power, some slurry within the barrel must sustain the maximum pressure (10,644.9 Pa) during printing. Higher slurry viscosity requires greater force from the driving mechanism to ensure consistent extrusion.

### 4.3. Comparative Analysis of Viscosity and Shear Rate Distribution

A propellant slurry is a pseudoplastic fluid, and forming complex propellant grains through extrusion 3D printing relies on the slurry’s strong shear-thinning properties. Insufficient shear thinning at the nozzle can lead to clogging, making it essential to study parameters such as shear behavior and viscosity distribution within the extrusion mechanism. This investigation helps to ensure smooth extrusion and improve the accuracy of 3D-printed structures.

**Figure 11:**
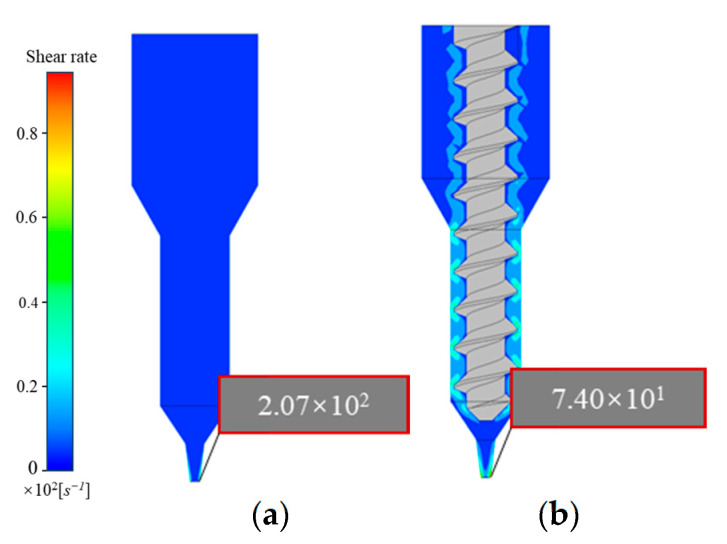
Shear rate distribution contour: (**a**) plunger extrusion printer; (**b**) screw extrusion printer.

**Figure 12:**
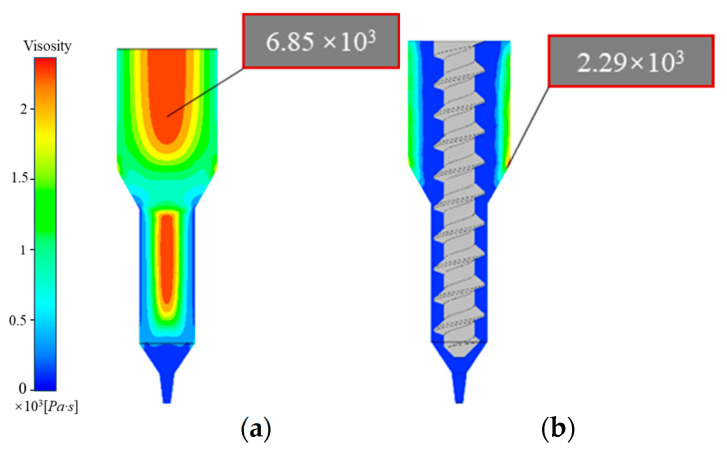
Viscosity distribution contour: (**a**) plunger extrusion printer; (**b**) screw extrusion printer.

As shown in Figure 11a, the shear rate within the plunger extrusion mechanism is relatively low and evenly distributed, nearing zero throughout the channel. The maximum shear rate occurs at the nozzle outlet, with values in the range of 102, showing a rapid increase from top to bottom. Compared to screw extrusion, the plunger mechanism exhibits a lower maximum shear rate (Figure 11b). These elevated shear rates near the nozzle outlet reduce the viscosity of the propellant slurry to a relatively low level (Figure 12a), allowing easy extrusion through narrow nozzle cross-sections.

Figure 11a,b indicate that screw extrusion produces higher and more complex shear rates within the flow channel than plunger extrusion. High shear rates are concentrated near the screw, with larger values observed between the threads and the wall in the transition section. During extrusion, the rapid rotation of the screw drives slurry movement, with the slurry in the gap experiencing high shear rates due to this rotational motion. This causes solid particles within the slurry to gradually shift toward the flow channel interior, rapidly reducing viscosity and potentially accelerating the screw rotation.

In Figure 12a,b, it is evident that, compared to the higher viscosity in plunger extrusion, the slurry in the screw extrusion flow channel exhibits lower viscosity and enhanced rheological behavior, facilitating flow. This provides an advantage for smoothly extruding high-solid-content, high-viscosity propellant slurries.

### 4.4. Comparative Analysis of Particle Transport in Slurry During Extrusion

**Figure 13:**
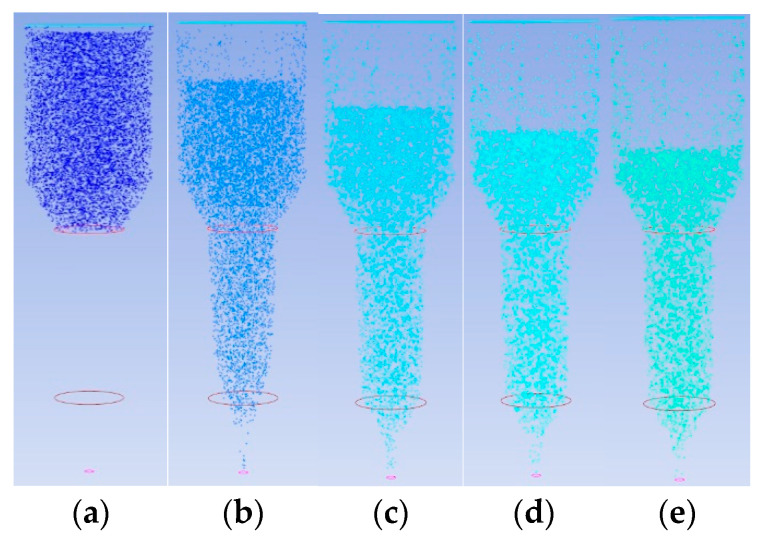
Particle distribution of plunger extrusion structure at different slices: (**a**) Slice 1; (**b**) Slice 8; (**c**) Slice 12; (**d**) Slice 16; (**e**) Slice 20.

**Figure 14:**
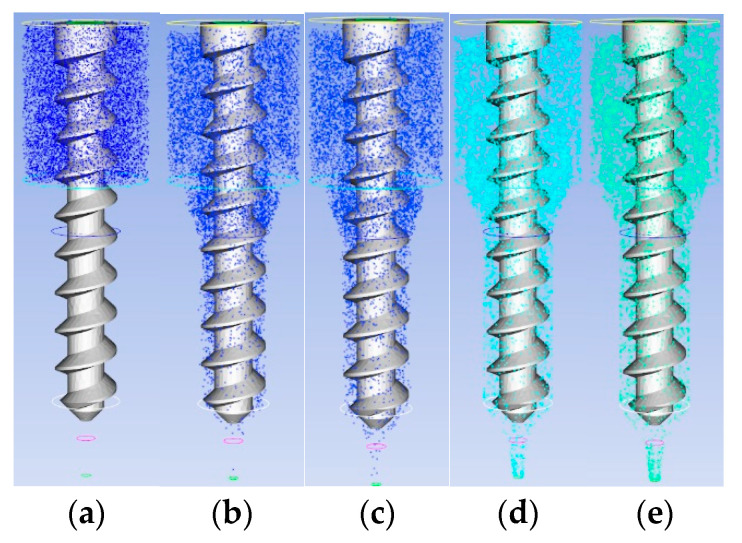
Particle distribution of screw extrusion structure at different slices: (**a**) Slice 1; (**b**) Slice 269; (**c**) Slice 300; (**d**) Slice 900; (**e**) Slice 1200.

To address particle transport issues in extrusion systems, a comparative analysis of particle transport in different extrusion structures was conducted using the hybrid simulation method in ANSYS/POLYFLOW 2022R1 software. Figure 13a and Figure 14b show the initial particle distributions within the two extrusion structures. In plunger extrusion, the propellant slurry fills the barrel by gravity after loading, so a particle source was established in the barrel section to simulate the initial extrusion state. For screw extrusion, the slurry is supplied continuously through an external feeder, with particle transport primarily driven by the rotation of the screw. Therefore, a particle source for screw extrusion was established within the cylindrical section of the barrel.

Figure 13a–e display particle distributions across different cross-sectional slices in the plunger extrusion flow channel. As the plunger compresses the slurry, particles gradually move toward the outlet, with particles starting to exit at the eighth slice. Between slices 12 and 20, most particles leave the barrel. Particle velocity increases as the particles travel from the barrel to the outlet, reaching a peak at the outlet. In contrast, Figure 14a–e show that in the screw extrusion mechanism, particles only begin to exit around the 269th slice, indicating that screw extrusion requires more time to reach a steady extrusion state when starting a new print job. Subsequently, the number of extruded particles increases, stabilizing from the 300th slice onwards as the screw rotates. However, by the 900th slice, some particles accumulate and stagnate near the outlet, resulting in a gradual reduction in particle extrusion and increasing the risk of nozzle clogging. Therefore, compared to the plunger-type mechanism, the screw-type extrusion requires a larger nozzle to ensure consistent slurry flow under identical extrusion conditions.

### 4.5. Verification of Extrusion-Forming Performance for Propellant Substitute Material

Using a custom-built extrusion 3D-printing platform, experiments were conducted with a composite solid propellant substitute slurry, using both plunger and screw extrusion processes to verify performance.

#### 4.5.1. Printing Parameter Setting and Sample Formation

The STL file of the sample was loaded into Cura 4.7 software, where relevant printing parameters were set, as shown in Table 5.

**Figure 15:**
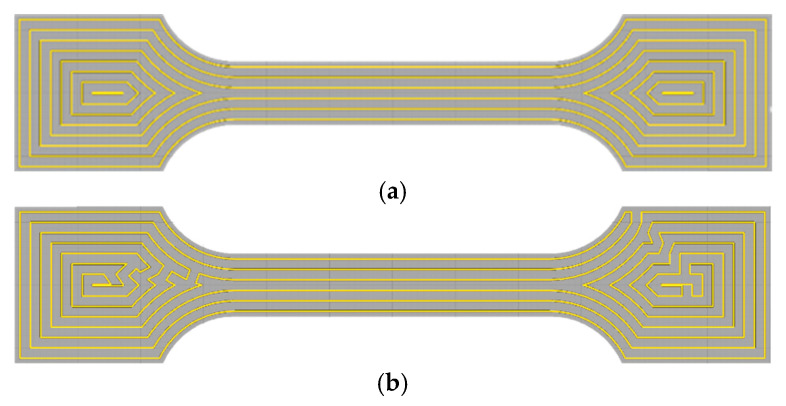
Path planning for dumbbell-shaped specimens: (**a**) non-connected concentric circle filling method; (**b**) connected concentric circle filling method.

Cura 4.7 offers various fill patterns, including zigzag, concentric circles, and straight lines [31]. To achieve a fully and tightly filled sample, a concentric circular filling strategy was selected, as shown in Figure 15a. However, with a concentric circle path, the nozzle must rapidly transition to the next circle after completing each pass. This can lead to overfilling at transition points due to the continuous flow of slurry; conversely, underfilling can occur even with retraction settings due to flow lag, resulting in printing defects. To address this, a connected concentric circle filling path was chosen, requiring only one continuous pass per layer. This eliminated the need for the nozzle to switch circles within each layer, thus enhancing the overall print quality.

**Figure 16:**
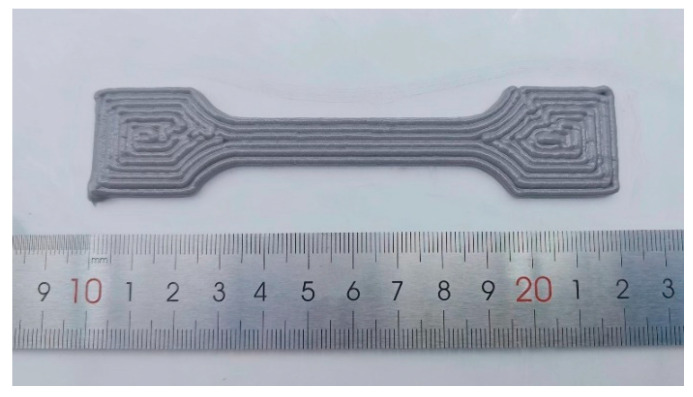
Dumbbell-shaped specimen printed using plunger extrusion process.

**Figure 17:**
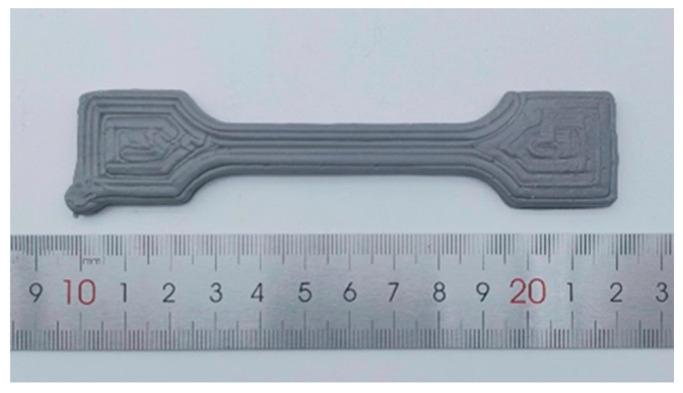
Dumbbell-shaped specimen printed using screw extrusion process.

After setting the parameters, slicing was performed, and the G-code file was generated. The extrusion 3D printer then executed the printing operations according to the G-code instructions. Based on the flow rate values established in the previous simulations, dumbbell-shaped specimens were formed using both plunger and screw extrusion methods, as shown in Figure 16 and Figure 17.

The resulting specimens are completely filled and free of visible defects, confirming that the specimens extruded at this flow rate were suitable for tensile testing. The apparent significant differences observed in the stress–strain curves are actually due to the small range selected for the *X*-axis values. Since the matrix resin used in this experiment is relatively brittle with almost no ductility, the range of *X*-axis values is small, leading to the stress–strain curves appearing to be quite different. We have supplemented the average and variance of the data from six specimens, and from a direct comparison of these numerical values, the differences are not very significant. Additionally, the printed propellant specimens have relatively low strength, and the sensitivity to how the specimens are clamped during testing is high. These factors can also contribute to fluctuations in the obtained data.

#### 4.5.2. Evaluation of Mechanical Properties

The mechanical properties of composite solid propellants are mainly influenced by three factors: the photosensitive resin, the solid filler, and the interfacial interaction between these components [32]. The photosensitive resin serves as the structural backbone of the composite solid propellant, with its mechanical properties largely determining those of the material. Although solid fillers, such as silica and aluminum powder, comprise over 80 wt% of the composite, the continuous phase of the photosensitive resin dictates the material’s mechanical behavior. Therefore, defects such as pores that arise from different forming processes and affect the bonding continuity between the resin and solid phase can further impact the mechanical properties of the specimens. Thus, the forming quality of each process is reflected in the resulting mechanical properties.

**Figure 18:**
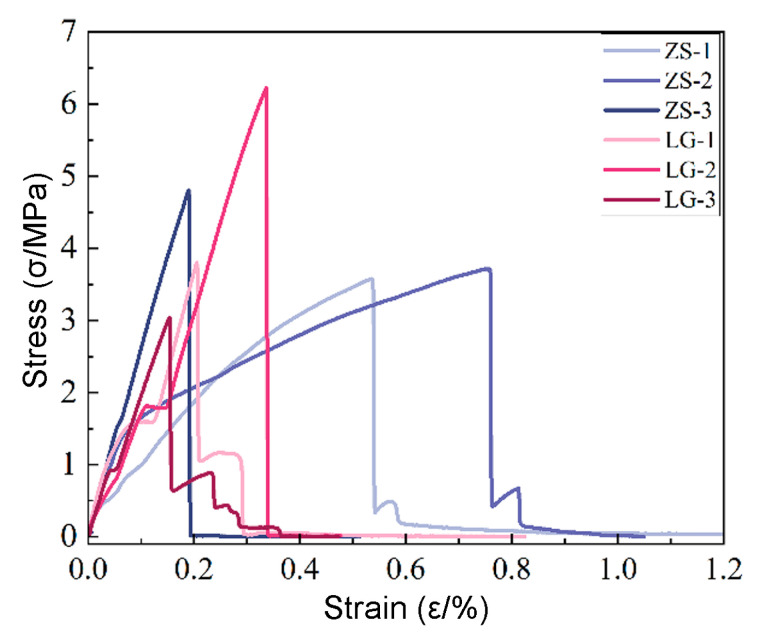
Stress–strain curves of specimens formed by screw and plunger extrusion.

Tensile tests were conducted on the printed propellant specimens using a universal tensile testing machine (INSTRON 5969, Instron, Norwood, NY, USA) at 20 °C with a tensile speed of 2 mm/min. The results, shown in Figure 18, depict the stress–strain curves of specimens formed by each method, where ZS13 corresponds to the specimens formed by plunger extrusion and LG13 corresponds to those formed by screw extrusion.

As shown in the figure, the specimens formed by both processes exhibit distinct hard and brittle mechanical characteristics mainly attributed to the binder properties (in this experiment, a photosensitive resin with higher brittleness was used solely for the purpose of comparing the screw and plunger processes, and there is a gap compared to the real propellant). The mechanical properties of the propellant specimens formed by each process are shown in Table 6. There was no significant difference in the tensile mechanical properties between the two specimen types, suggesting that further analysis, incorporating microscopic examination of the specimens after tensile failure, is needed to gain a deeper understanding.

#### 4.5.3. Microscopic Morphology

**Figure 19:**
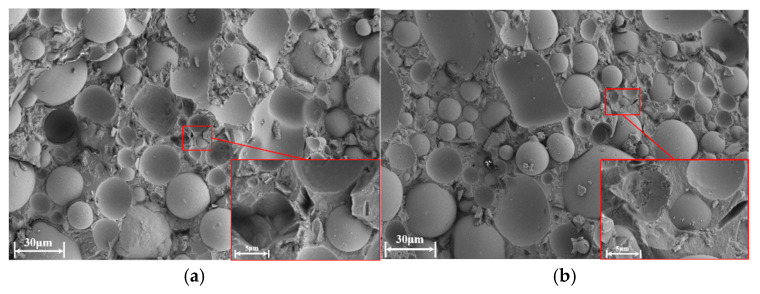
Fracture morphology of tensile specimens: (**a**) plunger extrusion; (**b**) screw extrusion.

The fracture morphologies of the tensile-fractured specimens, analyzed using scanning electron microscopy and shown in Figure 19, reveal relatively rough surfaces with smooth protrusions and pits left by the separation of solid filler particles from the cured photosensitive resin under stress, which is indicative of clear brittle fracture characteristics.

Both screw and plunger extrusion specimens displayed dense fracture morphologies. While some micro-sized pores were visible upon closer inspection, no large voids were observed. Based on fracture cross-section analysis alone, the forming quality of both processes appears nearly equivalent. However, to comprehensively assess pore defects, further analysis using CT scanning is necessary.

#### 4.5.4. Porosity Characterization

**Figure 20:**
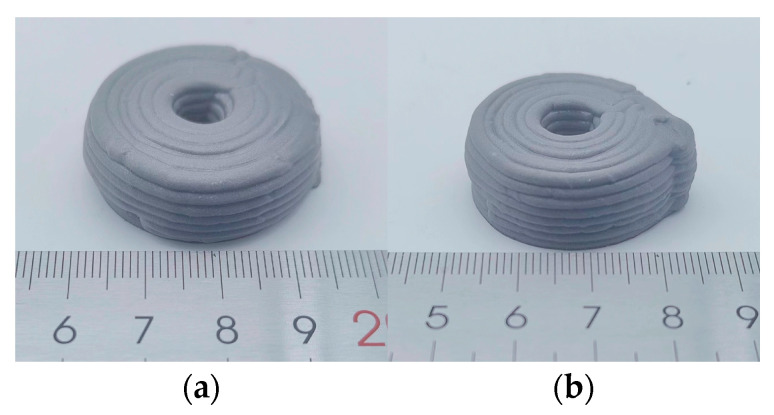
Substitute propellant grains: (**a**) plunger extrusion; (**b**) screw extrusion.

**Figure 21:**
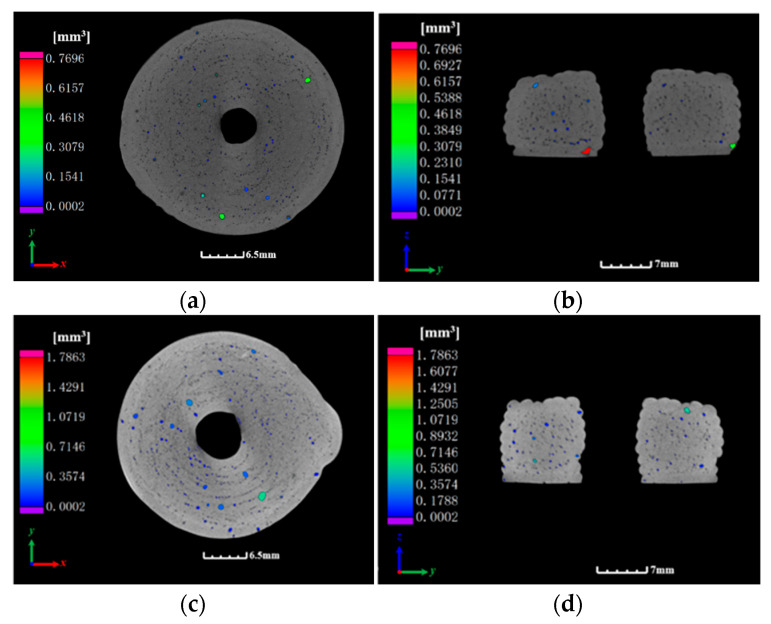
CT test results of substitute propellant grains: (**a**) radial cross-section of propellant grains formed by plunger extrusion; (**b**) radial cross-section of propellant grains formed by plunger extrusion; (**c**) radial cross-section of propellant grains formed by screw extrusion; (**d**) radial cross-section of propellant grains formed by screw extrusion.

To investigate potential differences in forming quality between the plunger and screw extrusion processes regarding the internal structure of the formed propellant grains, CT scanning was performed on circular propellant grains produced by each method. Figure 20 presents the external appearance of the propellant grains, while Figure 21 displays the CT scanning results, allowing for a detailed comparison of internal structural integrity.

The CT scan results in Figure 21 reveal that both screw and plunger extrusion processes produced pores and defects within the propellant grains, which were primarily located at the bottom and top of each printed layer. These pores are typically generated at the lap joints of the propellant paste lines during extrusion, while larger voids result from air bubbles incorporated during paste mixing. Overall, no significant differences were observed in defect distribution between the circular propellant grains formed by the two extrusion processes.

To quantitatively assess porosity, the porosities of propellant grains formed by each process were calculated, as shown in Table 7. The porosity of propellant grains formed by screw extrusion was 1.01%, whereas that of the plunger-extruded grains was 0.53%, indicating a slightly lower porosity in the plunger-extruded grains. The overall densities of the samples were 98.99% and 99.47%, respectively, showing only minor differences. Further analysis indicated that these defects were primarily concentrated in overlapping areas, arising from the extrusion-forming process—specifically, the overlaps between extrusion line units and interlayer joints—rather than from differences in extrusion technique.

Therefore, tensile testing, microscopic characterization, and CT scan results indicate that the mechanical properties and microscopic porosity of propellants formed by the plunger and screw extrusion processes are comparable.

This study utilized a combination of numerical simulations and experimental methods to investigate the extrusion process of propellant substitutes using plunger and screw extrusion, as well as to assess the mechanical properties and microscopic porosity of the formed propellant grains. These findings provide valuable insights for developing principles and methodologies for 3D-printing processes for propellants. In the future, our team will conduct in-depth research on in situ monitoring of the pressure field, velocity field, and particle distribution of propellant paste during extrusion to establish foundational theoretical and technological approaches for enhancing the stability and safety of the process.

## 5. Conclusions

Through numerical simulations and experimental research, this study investigated the extrusion process of propellant substitutes using plunger and screw extrusion, as well as the mechanical properties and microscopic porosity of the formed propellant grains. The following conclusions were drawn:

(1) Compared to screw extrusion, the plunger extrusion process exhibited simpler flow characteristics, with high velocity, high shear rate, and low-pressure distribution near the nozzle outlet, effectively facilitating smooth extrusion of the propellant paste. These characteristics were advantageous for extruding high-solid-content, high-viscosity solid propellants.
(2) The plunger extrusion process demonstrated a higher outlet velocity uniformity index (0.9276 > 0.8293) and more stable particle transport performance than the screw extrusion process, making it more suitable for high-precision propellant printing. In contrast, screw extrusion was more prone to propellant backflow, presenting potential safety risks.
(3) Propellant substitutes produced by both extrusion processes exhibited comparable mechanical properties and microscopic porosity, indicating similar performance in propellant grain formation across the two methods.

The numerical simulations were limited to the extrusion speed distribution and pressure drop, while the experiments focused on the mechanical properties and microstructural characterization of the printed propellant samples. The lack of direct integration between these two approaches is a limitation that will be addressed in future work. Furthermore, all experiments were conducted under controlled laboratory conditions, and the findings may not fully represent the complexities of industrial-scale applications.

## Figures and Tables

**Table 1:** Formula of substitute material of solid propellant.

Selection	Component	Function
SiO_2_	Substitute material for oxidant	Substitute for ammonium perchlorate (AP)
Al	Metal fuel	Release heat, increase energy level
Photosensitive resin	Binder	Bind solid filler particles and undergo photocuring reactions to form a three-dimensional network

**Table 2:** Proportioning of substitute material of solid propellant.

Solid Phase Content/wt%	SiO_2_/wt%		Al/wt%	Photosensitive Resin/wt%
	D_50_ = 235 μm	D_50_ = 5 μm	D_50_ = 33 μm	Polyurethane Acrylate (Self-Made)
84	60.25	13.75	10	16

**Table 3:** Parameter settings for simulation analysis.

Parameter Definition			Configuration
Material parameters of propellant slurry		g	9.8 m/s^2^
		Density	$1.89\;\times 10^{3}$ kg/m^3^
		Viscosity	Bird–Carreau
Screw settings	Boundary conditions	Inlet	*fn = fs =* 0
		Outlet	*fn = fs =* 0
		Wall	*vn = vs =* 0
	Setting of moving parts	Screw Speed	*n*
Plunger settings	Boundary conditions	Inlet	*fn = fs = 0*
		Outlet	*fn = fs = 0*

Note: *fn* refers to the normal force; *fs* refers to the shear force.

**Table 4:** Calculation of moving part parameters for both screw and plunger.

Structure	Formula for Calculation	Parameters	Parameters of Moving Parts
Plunger	$Q_{v}=\pi r_{out}^{2}v_{out}=\pi r_{in}^{2}v_{p}$	*Q_v_*, volume flow rate of slurry, mm^3^/s	*v_p_* = 0.26 mm/s
		*r_out_*, outlet cross- sectional radius, mm	
		*v_out_*, slurry outlet velocity, mm/s	
		*r_in_*, inlet cross- sectional radius, mm	
		*v_p_*, plunger movement speed, mm/s	
Screw	$Q_{f}=\frac{\pi }{2}whDn\cos\theta \cdot f$	*Q_f_*, approximate volume flow rate, mm^3^/s	*n* = 1 r/s
		*W*, screw width, *w =* 3 mm	
		*H*, thread depth, *h* = 2 mm	
		*D*, screw diameter, *D* = 10 mm	
		*n*, screw rotation speed, r/s	
		*θ*, thread angle, *θ* = 18.8°	
		*F*, fill ratio, 0.28	

**Table 5:** Printing parameter configuration.

Parameter Name	Setting
Layer height/mm	1.6
Line width/mm	1.6
Infill density/%	100
Infill line distance/mm	1.65
Print speed/mm·s^−1^	20
Infill pattern	Concentric circles

**Table 6:** Mechanical properties of composite solid propellant.

Process	Tensile Strength/MPa	Elongation at Break/%
Plunger extrusion molding	4.033	0.493
Screw extrusion molding	4.427	0.233

**Table 7:** The porosities of composite solid propellant.

Process	Material Volume/mm	Defect Volume/mm^−3^	Porosity/%
Plunger extrusion molding	9374.8	50.3	0.53
Screw extrusion molding	8352.0	85.3	1.01

## Data Availability

The original contributions presented in this study are included in the article. Further inquiries can be directed to the corresponding author.
